# Supplementary material for: Can the establishment of National Key Ecological Functional Zones improve air quality?: An empirical study from China
Source: PLoS One. 2021 Feb 16;16(2):e0246257. doi: 10.1371/journal.pone.0246257 (PMC7886127; doi:10.1371/journal.pone.0246257)
Supplement: Attachment — Submitted filename: Response to Reviewers-II.docx [file pone.0246257.s003.docx]

**Response to Reviewers**

First of all, we would like to thank reviewers for your insightful, constructive and helpful comments on our manuscript entitled “**Can the Establishment of National Key Ecological Functional Zones Improve Air Quality? —— An Empirical Study from China**”. We have carefully considered and addressed all the comments and made necessary revisions in the revised manuscript. We provide a point-by-point response to the reviewers’ comments below.

The points raised by the reviewers are written in **bold font,** whereas our responses are shown in normal font, and the quotation of the revised manuscript is shown in *italic font*.

# Reviewer #2:

**I read the paper. The paper has improved as compared to earlier version. I thank the authors for their effort. However, the paper can further be improved. The comments are listed below.**

***Response*:**

Thank you again for your valuable suggestions and opinions on the improvement of the paper, which plays an important role in improving the quality of the paper. We will continue to revise the paper at your suggestion.

**a) Although write-up has improved; the paper would benefit from one more detailed English edit for clarity and typos. For example; this sentence in line 96-97 can be improved “It is important to examine the effectiveness of the establishment of ecological functional zones in reducing air pollution because pollution has detrimental impacts on quality of life.” The last sentence reads “The problem will leave this for future research.”. I suggest a serious English edit.**

***Response*:**

Thank you for your suggestions on the English expressions in the manuscript. Indeed, there are many irregularities in our English expression. In the revised manuscript, we have asked English touch up company to make a comprehensive touch up of the voice, so as to meet the publishing requirements.

**b) The caption in figure 1 and Note needs revision. The author can follow the map caption in other papers.**

***Response*:**

Thank you for pointing out that the title and annotation of Figure 1 are not standard. We modify the caption and Note of Figure 1. Please see Pages 4-5 of the revised manuscript:

…

The difference-in-differences estimator was the independent variable (). Since the effect of the eco-functional zone policy put into effect in June 2011and has a time lag, 2012 is chosen to reflect the impacts of the policy. The green areas in Figure 1 represent the **NKEFZ**, which are the treatment group in this paper; the red areas are the control group which represent the **non-NKEFZ**. These are 436 county-level administrative regions of the more than 2000 counties in China which belong to the **NKEFZ**.

DID variables include the time dummy and the region dummy: the time dummy variables equal to 1 after the promulgation of the National Key Ecological Function Zones policy; the region dummy variables equal to 1 if the regions belong to the **NKEFZ**. Based on this, the interactive items between two dummy variables is DID variable. If the coefficient of the interactive items is negative and significant, it indicates that the air quality of the **NKEFZ** is improved.

**Fig 1. The map of the NKEFZ and the non-NKEFZ in China.**

*Notes:* The map is completed by ArcGIS software. The green areas are the treatment group which the counties belong to the NKEFZ, and the red areas is the control group which the counties belong to the non-NKEFZ. As some counties have no data, they are not included in the study sample, where these areas are shown in white on the map.

…

**c) In table-2: the author didn’t report the effect of the policy variable— ‘eco’ also. Is it intentionally removed? I suggest to include it.**

***Response*:**

Thank you very much for questioning the problems in the regression results. In the regression, the coefficient of *eco* is not reported. The reason is that in the regression, the fixed effect of time and fixed effect of region are controlled, which leads to the multi-collinearity of *eco* and time effect. As a result, the coefficient cannot be estimated (the estimated coefficient is 0), so the coefficient is not reported in the regression. In the revised manuscript, we modified the notes for each table.

**d) The Authors has explained the reason for not presenting the DID estimates in the figure. Their argument is the estimates and parallel trend test provides the same information. However, the use of the figure will make the result visible and easily understandable.**

***Response*:**

Thank you for your advice. In the revised manuscript, we made a parallel trend test figure based on the results of table 6, and revised the corresponding content of the paper. Please see Pages 4-5 of the revised manuscript:

…

The estimated results in columns (2), (4) and (6) of table 6 are made into Figure 2. It can be seen that after controlling for other factors, the three explained variables are significantly negative at the level of 5% after 2012, but not significant before 2011, which indicates that the three estimates meet the parallel trend test.

|  | lnavgPM | lnmedPM | lnmaxPM |
| --- | --- | --- | --- |
| eco*T2009 | -0.018 | -0.0112 | -0.0108 |
| eco*T2010 | -0.0153 | -0.0054 | -0.015 |
| eco*T2011 | 0.0002 | -0.0022 | 0.0107 |
| eco*T2012 | -0.1398 | -0.1351 | -0.1569 |
| eco*T2013 | -0.1713 | -0.1696 | -0.1756 |
| eco*T2014 | -0.1671 | -0.1652 | -0.1747 |
| eco*T2015 | -0.1889 | -0.1632 | -0.1786 |

Fig. 2 Parallel trend tests. Those figures are based on the estimated results in columns (2), (4) and (6) of table 6, with a confidence level of 5%.

…

**e) The author argues the result in Table -5 is comparable to the Main result in table-2. When 99% sample is matched, it is unsurprising to see the same result. The question is ‘What does 99% sample matched imply?’ Are the treated and Not-treated counties the same? and if yes on what basis. The author has to mention the variable used to match the sample. Also, because the Table-4 result varies widely for a different method of matching and with the main result.**

***Response*:**

Thank you for your question. Your question is very good. This is also the confusion we face in the process of writing. First of all, the sample loss after matching is less, only 1-2%, indicating that some samples do not match the treatment group, and these samples may be the year area with more serious pollution. Secondly, the estimated results of PSM-DID are much different from those of the main model. We think that it is because there are no matched samples that the pollution degree of these areas (counties) is more serious. Generally speaking, the counties that can become the NKEFZ are not the most serious in pollution. Therefore, those with better matching are not the ones with the most serious pollution. After removing the most polluted samples (the estimated coefficient becomes smaller), the difference between the treatment group and the control group is not so big, but it is still significantly negative, indicating that the effect of the policy still exists.

Finally, thank you for your suggestions. All of your suggestions are very important. They are of important guiding significance for my thesis writing and scientific research. We have done our best to revise the manuscript, but if any additional revision is needed, we will certainly do so under your directions.
